# Ultrasound assisted extraction and liposome encapsulation of olive leaves and orange peels: How to transform biomass waste into valuable resources with antimicrobial activity

**DOI:** 10.1016/j.ultsonch.2024.106765

**Published:** 2024-01-12

**Authors:** Giuliana Prevete, Loïc G. Carvalho, Maria del Carmen Razola-Diaz, Vito Verardo, Giovanna Mancini, Alberto Fiore, Marco Mazzonna

**Affiliations:** a Department of Chemistry and Technologies of Drug, Sapienza University of Rome, P.le Aldo Moro, 5, 00185 Rome, Italy; b CNR-Institute for Biological Systems (ISB), Area della Ricerca di Roma 1, Via Salaria Km 29,300, 00015 Monterotondo, Italy; c School of Applied Science, Division of Engineering and Food Science University of Abertay, Bell Street, DD1 1HG Dundee, Scotland, UK; d Department of Nutrition and Food Science, University of Granada, Campus of Cartuja s/n, 18071 Granada, Spain; e Institute of Nutrition and Food Technology ‘José Mataix’, Biomedical Research Centre, University of Granada, Avda. del Conocimiento s/n., 18100 Armilla, Granada, Spain

**Keywords:** Ultrasound assisted extraction, Olive leaves extract, Orange peels extract, Polyphenols, Liposomes, Antibacterial activity

## Abstract

Every year million tons of by-products and waste from olive and orange processing are produced by agri-food industries, thus triggering environmental and economic problems worldwide. From the perspective of a circular economy model, olive leaves and orange peels can be valorized in valuable products due to the presence of bioactive compounds such as polyphenols exhibiting beneficial effects on human health.

The aqueous extracts of olive leaves and orange peels rich in phenolic compounds were prepared by ultrasound-assisted extraction. Both extracts were characterized in terms of yield of extraction, total phenolic content and antioxidant capacity; the polyphenolic profiles were deeper investigated by HPLC-MS analysis.

Each extract was included in liposomes composed by a natural phospholipid, 1,2-dioleoyl-*sn*-glycero-3-phosphocholine, and cholesterol prepared according to the thin-layer evaporation method coupled with a sonication process.

The antimicrobial activity of the extracts, free and loaded in liposomes, was investigated according to the broth macrodilution method against different strains of potential bacterial pathogenic species: *Staphylococcus aureus* (NCIMB 9518), *Bacillus subtilis (*ATCC 6051) and *Enterococcus faecalis* (NCIMB 775) as *Gram*-positive, while *Escherichia coli (*NCIMB 13302), *Pseudomonas aeruginosa (*NCIMB 9904) and *Klebsiella oxytoca* (NCIMB 12259) as *Gram*-negative.

The encapsulation of olive leaves extract in liposomes enhanced its antibacterial activity against *S. aureus* by an order of magnitude.

## Highlights

• Olive leaves and orange peels extracts rich in polyphenols were prepared by ultrasound assisted extraction.
• Olive leaves and orange peels extracts were embedded in DOPC/Chol based liposomes prepared by sonication protocol.
• Ultrasounds provided liposomes with suitable physicochemical features, good entrapment efficiency and storage stability.
• The inclusion of olive leaves extract in sonicated DOPC/Chol liposomes enhanced its antimicrobial activity.

## 1 Introduction

Agri-food industries generate a high amount of by-products and waste, both solids and liquids, from the production, preparation and consumption of foods, representing a serious environmental and economic problem worldwide in terms of pollution, depletion of natural resources and compromised food safety [1]. Therefore, for the last decades it has been necessary to seek new strategies to transform biomass waste into valuable products, with the aim of minimizing waste production and obtaining biomaterials and compounds, which can deliver new solutions to existing problems. In this regard, a circular economy approach on agri-food wastes could represent an important opportunity to create sustainable growth and generate profit.

Citrus fruits and olives represent some of the main foods on which the Mediterranean diet is based, due to the high content of beneficial nutrients such as vitamins, minerals, and dietary fibers. The worldwide production of these two fruits counts for millions of tons per year and consequently high levels of waste and by-products are produced. In particular, in the orange juice industry orange peels often represent a waste whose annual production is estimated to be 32 million tons [2], whereas the pruning of olive trees in Europe generates 11.8 million tons of biomass [3].

Both these by-products represent a serious economic and environmental problem for producers. Meanwhile, they contain valuable and valued compounds produced by plants as secondary metabolites and known as phytochemicals [4].

Polyphenols are the major group of bioactive compounds present in citrus peels and olive leaves, which are ubiquitously distributed in all higher plants and have an important role as defence against plant pathogens and as response to different abiotic stress conditions [5]. Polyphenols exhibit many positive effects on well-being due to their antioxidant [6], [7], antimicrobial [8], [9], anti-inflammatory [10], anti-atherogenic [11] and anticancer [12] properties; thanks to their properties they have gained pivotal attention in many application fields [13].

Polyphenols extracted from vegetable foods and plants have been extensively investigated in the last 30 years for their potential antimicrobial activity against a wide range of bacteria, both *Gram*-positive and *Gram*-negative [5], [14], [15]. In particular, olive leaves extracts (OLEs) have demonstrated to be active against a wide range of bacteria, including *Listeria monocytogenes*, *Escherichia coli*, *Klebsiella pneumoniae*, *Staphylococcus aureus*, *Yersinia enterocolitica*, *Salmonella typhi*, *Bacillus cereus*, *Bacillus subtilis*, *Pseudomonas aeruginosa*, *Helicobacter pylori*, *Vibrio parahaemolyticus*, *Campylobacter jejuni*, and *Candida albicans* [16], [17], while orange peels extracts (OPEs) have been found to have antimicrobial activity against *Staphylococcus aureus*, *Enterococcus faecalis*, *Pseudomonas aeruginosa*, *Escherichia coli*, *Salmonella typhimurium*, *Listeria innocua*, *Bacillus cereus, Listeria monocytogenes Salmonella senftenberg*, and *Yersinia enterocolitica* [18], [19], [20]*.*

Phenolic compounds present in olive leaves [21] and orange peels [22] can be extracted according to different procedures such as conventional solvent extraction [23], supercritical fluid extraction [24], microwave-assisted extraction [25] or ultrasound-assisted extraction [26]. Among these techniques, ultrasound-assisted extraction (UAE) is widely recognized as a green and innovative procedure, because it involves reduced operations and relatively low costs, moderate energy consumption and short processing time; in addition, low quantity of water and solvents are generally required [27]. UAE is based on the principle of acoustic cavitation capable of damaging the cell walls of the vegetal matrix thus favouring the release of bioactive compounds through several mechanisms, such as the collision between particles and ultrasonic waves or the implosion of bubbles solvent on the surface of the vegetal matrix [28].

However, most of natural compounds have shown low bioavailability because of intrinsic factors (chemical structure, low water solubility) and extrinsic factors (low stability in biological fluids, extensive phase 1 and phase 2 metabolism, rapid elimination), high sensitivity to environmental conditions (temperature, pH, light, presence of oxygen, enzymatic activity) and poor sensorial characteristics, thus preventing their potential use. In order to improve bioavailability a number of nano-encapsulation techniques have been developed [29]. Among various delivery systems, liposomes have shown promising advantages as carriers of bioactive agents owed to their ability to encapsulate hydrophilic and hydrophobic compounds, enhanced paracellular and transcellular cargo transport, and their low toxicity and biodegradable nature [30].

Liposomes are phospholipid-based vesicles composed of one or more lipid bilayers enclosing internal aqueous compartments. Due to their nature, liposomes are able to simulate the behavior of cell membranes and have been recognized by the pharmaceutical industry as a formidable tool to treat different diseases and address several therapeutic issues [31], [32]. They have been applied for many medical purposes, such as in anticancer therapy, vaccination, gene therapy, pulmonary treatment, eye treatment and diagnostics [31], [32].

The versatility possessed by liposomes has allowed to successfully convey many pharmaceutical substances (antibiotics, antifungals, anti-inflammatory drugs, etc.) as well as plant extracts (Callendula officinalis, Dracocephalum moldavica, etc.) [32].

The application of liposomes as a delivery system could, potentially, enhance or reduce the biological activity of the conveyed substances [33]. For example Faezizadeh et al. reported a four-fold increase in antibacterial efficacy of *Silybum marianum* extract against MRSA after encapsulation in liposomes formulated with egg lecithin and cholesterol (MIC of 500 mg/L for unloaded extract and 125 mg/L for extract loaded liposomes) [34], as well as Karimi et al. reported an increased antimicrobial activity of turmeric extract encapsulated in liposomes (formulated with phosphatidylcholine) compared to that of the free extract against different bacteria species [35]. For those substances that may possess several biological activities, the encapsulation in liposomes can even increase some of these properties and suppress others, as it was found for the encapsulation of *trans*-resveratrol (a stilbenoid polyphenol synthesized by seventy-two different plant species) in liposomes functionalized with galactosylated amphiphile, where *trans*-resveratrol anti-adhesive and anti-biofilm properties against *S. aureus* and MRSA were greatly amplified after encapsulation while its bacteriostatic properties was completely knocked down [36].

Liposomes can be prepared by sonication technique, a simple green method widely exploited since the 1960 s [37]. Sonication acoustic energy is employed to convert large and multilamellar vesicles or vesicle aggregates in smaller unilamellar liposomes, either empty or loaded with a cargo. The effect on the reduction of sizes, lamellarity and polydispersity index are closely related to the methodology specifications such as sonication power and sonication time [38], [39] and can be ascribed to the cavitation phenomena [40]*.* Probe and bath sonication are the two main sonication methods used in liposomes production, besides probe sonication is probably the most widely used method of the two for the preparation of liposomes on small scale, because the sample has not to be warmed above the phase transition temperature due to local heating, and the high energy input can be applied directly into the lipid dispersion to obtain vesicles with suitable features.

Here we report on an investigation aimed at evaluating the effect of the encapsulation in liposomes on the *in vitro* antimicrobial activity of olive leaves and orange peels extracts against different strains of potential bacterial pathogenic species, in particular *Staphylococcus aureus* (NCIMB 9518), *Bacillus subtilis (*ATCC 6051) and *Enterococcus faecalis* (NCIMB 775) as *Gram*-positive bacteria, and *Escherichia coli* (NCIMB 13302), *Pseudomonas aeruginosa* (NCIMB 9904) and *Klebsiella oxytoca* (NCIMB 12259) as *Gram*-negative bacteria.

The best ultrasound-assisted extraction conditions using a sonotrode were established to obtain polyphenols-rich extracts, which were characterized in terms of yield of extraction, total phenolic content and antioxidant capacity. The polyphenolic profiles of extracts were investigated by HPLC–ESI-TOF-MS analysis.

**Fig. 1:**
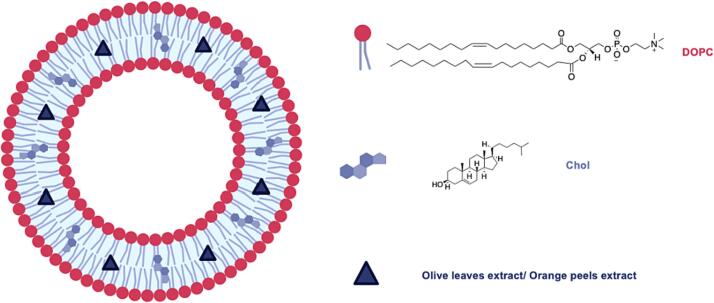
Schematic representation of liposomes including vegetal extracts (created with BioRender.com).

Liposomes formulated with a natural phospholipid, namely 1,2-dioleoyl-*sn*-glycero-3-phosphocholine (DOPC) and cholesterol (Chol) and including olive leaves and orange peels extracts (Fig. 1) were characterized in terms of particle features, encapsulation efficiency, stability and releasing profile over time.

## 2 Materials and methods

### 2.1 Materials

Olive leaves from *Olea europaea* and orange peels from *Citrus sinensis* were provided by Bidah–Chaumel (Lorquì, Murcia, Spain) as dry materials. Gallic acid (purity 97 %), trolox ((±)-6-hydroxy-2,5,7,8-tetramethylchromane-2-carboxylic acid (purity ≥ 97 %), trichloroacetic acid, hydroxytyrosol, oleuropein, apigenin-7-glucoside, rutin, luteolin, vanillic acid, quercetin, chlorogenic acid, ferulic acid, Folin & Ciocalteu′s phenol reagent, phosphate-buffered saline (PBS; 0.01 M phosphate buffer, 0.0027 M KCl, 0.137 M NaCl, pH 7.4, at 25 °C), cellulose dialysis membrane (D9527-100FT, molecular weight cut off = 14 kDa), sodium carbonate (purity ≥ 98 %), cholesterol (purity 99 %), chloroform, methanol, ethanol, acetic acid, acetonitrile, and water (all HPLC grade) were purchased from Sigma-Aldrich, UK. 1,2-dioleoyl-*sn*-glycero-3-phosphocholine (DOPC) was purchased from Avanti Polar Lipids (Alabaster, AL, USA). ABTS (2,2′-Azino-bis (3-ethylbenzothiazoline-6-sulfonic acid) diammonium salt, purity ≥ 98 %) and potassium persulfate were purchased from Roche Diagnostic GmbH, UK. DPPH (2,2-diphenyl-1-picrylhydrazyl, purity 95 %) was purchased from Alfa Aesar, UK. Iron (III) chloride hexahydrate (purity 97 %), Muller Hinton Broth (CM 0405) and Muller Hinton Agar (CM 0337) were purchased from Thermo Fisher Scientific, UK.

### 2.2 Ultrasound-assisted extraction

#### 2.2.1 Olive leaves extracts (OLE)

Olive leaves extracts were obtained by ultrasound-assisted extraction, using a UIP2000hdT (20KHz, 2000 W) ultrasonicator (Hielschier, Germany) settled with the ultrasound generator, transducer and radial sonotrode (RS4d40L4, d = 40 mm) in a batch process. Dried olive leaves were grinded to a fine powder that was suspended into a cylinder filled with chilled water (4–6 °C), at 1:50 (w/v) sample:water ratio. The cylinder was immersed in an ice bath to keep temperature below 75 °C during sonication process. The extraction process was carried out taking into consideration the influence of the extraction time (from 5 to 25 min) and acoustic parameters (amplitude, total power (W), energy transferred (Ws) and power density (Ws/mL)) on the yield of extraction and on the total phenolic content. All the obtained extracts were filtered with a strainer and centrifuged (Hermle Z323K, UK) at 8000 rpm at 4 °C for 15 min. Finally, the supernatants were protected from light and stored under refrigeration (–20 °C) until spray drying process.

#### 2.2.2 Orange peels extracts (OPE)

Orange peel extract was produced at Bio Based Europe Pilot Plant on a pilot scale trial process in the framework of the European Project Shealthy (Horizon 2020 - grant number 817936). 70 kg of powdered dried orange peels in 600 L of water were enzymatically treated using 400 mL of Pectinex ULTRA SP-L at 30 °C for 24 h. Afterwards, the ultrasound assisted extraction was performed by an UIP2000hdT (20KHz, 2000 W) ultrasonicator (Hielschier, Germany) apparatus settled with the ultrasound generator, transducer and cascatrode (CS4d40L3, d = 40 mm). The slurry was recirculated over sonicator at 10 °C for 12 h. The residual solid matrix was removed via decanter, while the liquid extract was subjected to different filtration processes: microfiltration (0.45 μm), ultrafiltration (10 kDa), nanofiltration (0.15–0.30 kDa) and sterile filtration (PES 0.2 μm). Finally, the extract produced was stored at −20 °C until spray drying process.

### 2.3 Spray-Drying process

250 mL aliquots of each extract were spray dried by a Büchi Mini Spray Dryer B-290, using the following parameters: inlet temperature 170–180 °C, aspirator 100 %, pump 20 %, flow 40–60 %. For each sample, the yield of extraction was determined as the percentage ratio between the weight of the dry extract residue and that of the plant material used in the extraction process **Eq.** (1):

(1) $$R\left(\%\right)=\frac{g_{\mathrm{spray}-dried\;extract}}{g_{\mathrm{plant}\;\mathrm{material}}}\times 100$$

The final spray-dried extracts were stored at −20 °C before use.

### 2.4 Total phenolic content

The Total Phenolic Content (TPC) of the extracts was determined by Folin-Ciocalteu assay, following the procedure reported by de Falco et al [41].

Briefly, Folin-Ciocalteu (FC) reagent was diluted with water (1/10 v/v) and protected from light; then 540 µL of diluted FC reagent and 432 µL of 7.5 % (w/v) Na_2_CO_3_ solution were added to 27 µL of sample (concentration ranging from 0.4 mg/mL to 10 mg/mL) and incubated at 50 °C for 5 min. Finally, the absorbance was measured at 760 nm using a spectrophotometer (Thermo GENESYS^TM^ 10UV UV–Vis) and readings were performed in triplicate. The total phenolic content was calculated using Gallic Acid as reference standard (calibration curve 15.3–500 µg/mL) and expressed in milligrams of Gallic Acid equivalents (GAE) per gram of dry extract (mg_GAE_/g_extract_).

### 2.5 Determination of antioxidant capacity

#### 2.5.1 Trolox Equivalent antioxidant capacity (TEAC assay)

TEAC assay was used to evaluate the antioxidant capacity of OLE and OPE according to the procedure reported by de Falco et al [41].

Briefly, ABTS radical cation (ABTS^•+^) was generated by reacting 7 mM ABTS and 140 mM potassium persulfate leaving the solutions under stirring overnight at 4 °C in the dark, then the aqueous solution of ABTS^•+^ was diluted to obtain an absorbance of 0.700–0.750 at 734 nm. 1 mL of ABTS^•+^ solution was then added to 100 µL of sample (concentration ranging from 0.4 mg/mL to 2.0 mg/mL). The mixture was kept at room temperature for 150 s and the absorbance was measured at 734 nm.

Readings were assessed in triplicate and were used to determine the % of inhibition according to the following equation **Eq.** (2):

(2) $${\%}_{inhibition}=\left(1-\frac{{\mathrm{Abs}}_{\mathrm{sample}}}{{\mathrm{Abs}}_{\mathrm{control}}}\right)\times 100$$

where Abs_sample_ is the absorbance of the sample in the presence of ABTS and Abs_control_ is the absorbance of ABTS^•+^ solution.

Trolox, a water-soluble analogue of vitamin E, was used as reference standard and a calibration curve (3.90–62.6 µg/mL) was made plotting the percentage of ABTS^•+^ inhibitions as a function of micrograms (μg) of Trolox added.

% of inhibition of extract samples were finally expressed as milligrams of Trolox equivalents (TE) per gram of dry extract (mg_TE_/g_extract_).

#### 2.5.2 DPPH radical-scavenging assay

The scavenging activity of OLE and OPE on DPPH free radical was measured according to the following procedure [42], [43]. 1 mM DPPH stock solution in methanol was prepared and diluted with methanol to obtain a DPPH working solution characterized by an absorbance of 0.800–0.900 at 517 nm. Extract analyses were carried out by adding 1 mL DPPH working solution to 20 µL of extract (concentration ranging from 2 mg/mL to 10 mg/mL). The mixture was incubated 10 min at room temperature and the absorbance was measured at 517 nm. All analysis were carried out in triplicate and the percentage of inhibition was calculated as reported in equation **Eq.** (3):

(3) $${\%}_{inhibition}=\left(1-\frac{{\mathrm{Abs}}_{\mathrm{sample}}}{{\mathrm{Abs}}_{control}}\right)\times 100$$

where Abs_sample_ is the absorbance of the sample in the presence of DPPH and Abs_control_ is the absorbance of the DPPH solution.

Gallic Acid was used as reference standard and a calibration curve (5.0–150 µg/mL) was made plotting the percentage of DPPH inhibitions as a function of μg of Gallic Acid added.

% of inhibition of extract samples were finally expressed as milligrams of Gallic Acid equivalents (GAE) per gram of dry extract (mg_GAE_/g_extract_).

#### 2.5.3 Ferric reducing ability power

Antioxidant capacity of OLE and OPE was also assessed evaluating their ferric reducing ability, following the procedure reported by Benzie et al., slightly modified [44]. In particular, i) a 300 mM sodium acetate buffer solution, adjusted to pH 3.6 with acetic acid, ii) a 10 mM ferrous-TPTZ (2,4,6-tris(2-pyridyl)-s-triazine) complex solution in 40 mM HCl, and iii) a 20 mM FeCl_3_·6H_2_O solution were prepared. FRAP reagent was prepared by mixing 25 mL of sodium acetate buffer with 2.5 mL of ferrous-TPTZ solution and 2.5 mL of FeCl_3_·6H_2_O solution. To perform the assay, 900 µL of FRAP reagent were added to 100 µL of sample (ranging from 0.2 mg/mL to 2 mg/mL) and the mixture was allowed to react for 4 min at room temperature. The absorbance was then measured at 517 nm in triplicate. Gallic Acid was used as reference standard (calibration curve 0.025–0.40 µg/mL) and results were expressed as milligrams of Gallic Acid equivalents (GAE) per gram of dry extract (mg_GAE_/g_extract_).

### 2.6 Determination of OLE phenolic profile by HPLC-ESI-TOF-MS analysis

The phenolic composition of OLE was determined according to the method previously described by Talhaoui et al. slightly modified [45], [46]. The equipment consists of an ACQUITY (Water Corporation, Milford, MA, USA) UPLC system coupled with a time-of-flight analyzer (TOF) (Water Corporation, Milford, MA, USA). Phenolic compounds were separated by a Poroshell 120 EC-C18 analytical column (4.6 x 100 mm, 2.7 mm) from Agilent Technologies, under the following conditions: column temperature 25 °C, flow rate 0.8 mL min^−1^, 2.5 µL injection volume. The mobile phases were water with 1 % acetic acid (phase A) and acetonitrile (phase B), changing the solvent gradient as it follows: 0 min, 5 % B; 4 min, 9 % B; 7 min, 12 % B; 8 min, 15 % B; 9 min, 16 % B; 14 min, 20 % B; 15 min, 22 % B; 18 min, 28 % B; 19 min, 30 % B; 20 min, 31 % B; 24 min, 40 % B; 28 min, 100 % B; 31 min, 100 % B; 33 min, 5 % B. Mass spectrometer was equipped with an interface with electrospray ionization (ESI) source operating in negative mode. Operational conditions were: capillary voltage, 2300 kV; source temperature, 100 °C; cone gas flow, 40 L/h; desolvatation temperature, 500 °C; desolvatation gas flow, 11.000 L/h; scan range, *m*/*z* 50–1500. MassLynx 4.1 (Water Corporation, Milford, MA, USA) software was used to process acquired data.

Phenolic compounds were identified according to their *m*/*z* molecular formula and by comparing them with data reported in the literature [47] and with several databases (PubChem, KEGG COMPOUNDS Database), and by the co-elution with commercial standards, when possible.

The quantification of phenolic compounds in the extracts was performed by using five different standards, namely, hydroxytyrosol, apigenin-7-glucoside, rutin, luteolin and oleuropein. Their calibration curves were assessed in the range of 1–250 µg/mL at eight concentrations. Analyses were performed in duplicate.

### 2.7 Determination of OPE phenolic profile by HPLC-ESI-TOF-MS analysis

The analyses on OPE were assessed according to the procedure previously stated by Verni et al [48]. The analysis was carried out by an ACQUITY UPLC system (Waters Corporation, Milford, MA, United States) coupled to an electrospray ionization (ESI) source operating in the negative mode and a time-of-flight (TOF) mass detector (Waters Corporation, Milford, MA, United States) following these conditions: capillary voltage, 2300 kV; source temperature, 100 °C; cone gas flow, 40 L/Hr; desolvatation temperature, 500 °C; desolvatation gas flow, 11,000 L/h; scan range, *m*/*z* 50–1500. The compounds of interest were separated on an ACQUITY UPLC BEH Shield RP18 column (1.7 µm, 2.1 mm x 100 mm; Waters Corporation, Milford, MA, United States) at 40 °C. The elution gradient was carried out using water containing 1 % acetic acid (phase A) and acetonitrile (phase B), and applied as follows: 0 min, 1 % B; 2.3 min, 1 % B; 4.4 min, 7 % B; 8.1 min, 14 % B; 12.2 min, 24 % B; 16 min, 40 % B; 18.3 min, 100 % B, 21 min, 100 % B; 22.4 min, 1 % B; 25 min, 1 % B. The sample volume injected was 2 μL and the flow rate used was 0.6 mL/min. The compounds were monitored at 280 nm. Integration and data elaboration were performed using MassLynx 4.1 software (Waters Corporation, United States). For the quantification of phenolic compounds, solutions of ferulic acid, chlorogenic acid, vanillic acid, catechin, rutin and quercetin in methanol:water 1:1 v/v were prepared and used as standards. The calibration curves were elaborated by using the peak areas of each standard measured by HPLC at different concentrations from LOQ (0.14–1.57 µg/mL) to 250 µg/mL.

### 2.8 Preparation of liposomes

Liposomes, both empty and extract loaded, were formulated with a natural unsaturated phospholipid (DOPC, 6.28 mg/mL) and cholesterol (Chol, 0.77 mg/mL). Empty and loaded liposomes were prepared according to the Thin-Layer Evaporation method combined with the sonication protocol reported below [49]. In particular, the proper amount of lipid components (DOPC and Chol) was dissolved in chloroform, while the dried extracts (OLE or OPE, 7.05 mg/mL) were dissolved in methanol to obtain a final ratio lipids:extract 1:1 (w/w). All the components were mixed in a round bottom flask, dried by rotary evaporation and then under a flux of nitrogen to remove all trace of solvents and obtain a thin lipid film, which was hydrated with a phosphate buffer saline solution (PBS 150 mM) to give a 10 mM in total lipids concentration (DOPC 8 mM and Chol 2 mM), then vortex-mixed to completely detach the film from flask wall. The resulting multilamellar vesicles were freeze-thawed five times from liquid nitrogen to 50 °C and then were subjected to 15 min of sonication (Model Q55, Sonica Sonicator) in pulsed mode (3 min ON and 3 min OFF) at an amplitude of 20 % of full power. The round bottom flask containing the sample was immersed in an ice/water bath to avoid the degradation of the sample due to the local overheating resulting from energy dissipation at the sonicator tip [49]. Finally, to remove the metallic particles resulting from tip erosion and the larger lipid particles, the suspensions were centrifuged at 14.000 rpm for 10 min. The removal of unentrapped extract was performed by dialysis in PBS (buffer volume 25-times the total volume of the sample) by changing the diffusate buffer every 30 min over 2 h and keeping the system slowly stirred throughout.

### 2.9 Physicochemical characterization of liposomes

#### 2.9.1 Size and ζ-potential measurements

A Zetasizer Nano ZS (Malvern Instruments) equipped with a 5 mV He/Ne laser (λ = 632.8 nm) was used to measure size distributions, polydispersity index (PDI) and ζ-potential of samples. Temperature was set at 25 °C in all the measurements carried out.

Particle size and polydispersity index (PDI) were determined through the backscatter detection at an angle of 173°. The measured autocorrelation function was analysed by using the cumulant fit. The first cumulant was used to obtain the apparent diffusion coefficients (D) of the nanoparticles, further converted into apparent hydrodynamic diameters (D_h_) by using Stokes-Einstein relation **Eq.** (4):

(4) $$D_{h}=\frac{K_{b}T}{3\pi \eta D}$$

where k_B_T is the thermal energy and η is the solvent viscosity.

Before the measurements, suspensions of liposomes were diluted to 1 mM in total lipid concentration in PBS (150 mM) and then analysed by DLS.

The ζ-potential of liposomes was determined from the electrophoretic mobility (μ). Low voltages were applied to avoid the risk of Joule heating effects. Analysis of the Doppler shift to assess the electrophoretic mobility was done by using phase analysis light scattering (PALS) [50], a method which is especially useful at high ionic strengths, where mobility is usually low. The mobility μ of the liposomes was converted into a ζ-potential using the Smoluchowski relation ζ = μ η/ε, where ε and η are the permittivity and the viscosity of the solution, respectively. Liposomes were diluted to 1 mM in total lipid concentration in diluted PBS (15 mM).

All data reported of hydrodynamic diameter, PDI and ζ-potential correspond to the average of three different measurements.

#### 2.9.2 Evaluation of liposomes stability

The stability of extract loaded and empty liposomes was evaluated by checking vesicles size and PDI up to 28 days of storage at 4 °C, protecting samples from light sources. Measurements were performed as described in the above section.

#### 2.9.3 Determination of the Entrapment efficiency

The Entrapment Efficiency (EE%) of OLE and OPE into liposomes was determined by Folin-Ciocalteu assay. In particular, the content of total phenolic compounds was assessed on the extracts loaded into liposomes and compared with the amount measured in the spray dried extracts. The suspensions of liposomes were properly diluted with methanol (1:1 v/v) to break lipid aggregates thus triggering the release of loaded phenolic compounds. The assay was carried out also on empty liposomes diluted with methanol (1:1 v/v) to assess the contribution to the Folin-Ciocalteu assay due to lipid components. Absorbance was measured at 760 nm and readings were performed in triplicate. The results were expressed as micrograms of Gallic Acid equivalents (μg_GAE_).

Finally, the entrapment efficiency was calculated as follows (**Eq.** (5)):

(5) $$\mathrm{EE}\%=\frac{\;{\left(\mu g_{\mathrm{GAE}}\right)}_{\mathrm{loaded}\_\mathrm{liposome}}-{\left(\mu g_{\mathrm{GAE}}\right)}_{\mathrm{empty}\_\mathrm{liposome}}}{{\left(\mu g_{\mathrm{GAE}}\right)}_{\mathrm{dry}\_\mathrm{extract}}}x100$$

where (μg_GAE_)_loaded_liposome_, (μg_GAE_)_empty_liposome_ and (μg_GAE_) _dry_extract_ are respectively the micrograms of gallic acid equivalents obtained for extract loaded liposomes, empty liposomes and spray dried extract.

#### 2.9.4 *In vitro* release of extracts from liposomes

The release of phenolic compounds from OLE and OPE loaded liposomes was determined by dialysis method (PBS volume 50-times the total volume of the sample). Samples were collected every 1 h over a period of 24 h and analysed by Folin-Ciocalteu assay (Gallic Acid used as reference standard, calibration curve 10–2000 μg/mL) to study the releasing profile of the polyphenols encapsulated. All the collected liposomal aliquots were analysed after dilution with methanol (1:1 v/v) to break the lipid aggregates and to enhance the release of phenolic compounds entrapped. Then, the assay was assessed as described above. The phenolic content still encapsulated in liposomes was determined at a specific time and expressed as micrograms of Gallic Acid equivalents per mL (μg_GAE_/mL).

### 2.10 *In vitro* antimicrobial activity

#### 2.10.1 Bacterial strains

Antimicrobial activity assessment of OLE and OPE, both free and loaded in liposomes, was evaluated against different bacteria strains: *Staphylococcus aureus* (NCIMB 9518), *Bacillus subtilis (*ATCC 6051) and *Enterococcus faecalis* (NCIMB 775) as *Gram*-positive, as well as *Escherichia coli (*NCIMB 13302), *Pseudomonas aeruginosa (*NCIMB 9904) and *Klebsiella oxytoca* (NCIMB 12259) as *Gram*-negative.

#### 2.10.2 Determination of Minimum Inhibitory concentration (MIC) and Minimum Lethal concentration (MLC)

The broth macrodilution method was used to measure quantitatively the *in vitro* antimicrobial activity of OLE and OPE, both in free form and loaded in liposomes, against the selected bacteria strains. As described in the Clinical and Laboratory Standards Institute (CLSI) guidelines [51], an overnight culture of each bacterial strain was prepared in Muller Hinton Broth (MHB) and incubated at 37 °C. The bacterial inoculum was then prepared by dilution in MHB by adjusting the turbidity of the suspension in order to reach an optical density comparable to that of a 0.5 McFarland standard solution, which corresponds to a suspension containing approximately 1–2 x 10^8^ CFU/mL.

Furthermore, a solution of the extract, in free form or loaded in liposomes, was prepared and serially diluted in MHB.

Finally, a series of 10 test tubes was filled with 1 mL of the bacterial inoculum and 1 mL of the extract solutions, incrementally increasing the concentration of the extract in the tubes (0.10 mg/mL – 10 mg/mL). All tubes were mixed and incubated at 37 °C for 24 h. Minimum Inhibitory Concentration (MIC) was deduced from the first tube of the series where bacterial growth did not occur (no turbidity, no deposit of bacterial products). Growth inhibition in each test tube was compared to the growth control (positive control, free treatment test tube). The test tube in which bacterial growth was not detected were streaked on Muller Hinton Agar (MHA) plates, which were then incubated at 37 °C for 24 h. Finally, the Minimum Lethal Concentration (MLC) was deduced from the lowest concentration at which no culture was observed on MHA plates. The experiments were repeated until three consistent results were achieved.

### 2.11 Statistical analysis

IBM SPSS Statistics Version 23.0, Armonk, NY: IBM Corporation software was utilized for statistical analysis of the obtained data.

Significant statistical differences (p < 0.05) in TPC, yield of extraction (%), particle size diameter (D_h_) and PDI were analysed using a one-way ANOVA test. Post hoc analysis was performed via the Tukey’s HSD test to assess differences between the categories with a confidence interval of 95 %. Means were considered significantly different at p < 0.05. All the data were presented in the present study in the form of mean with the standard deviation (SD). The average was calculated using the results of the three treatment (biological) replicates and the three technical replicates (nine observations per sample).

## 3 Results and discussion

### 3.1 Preparation and characterization of extracts

The optimization of UAE of olive leaves was tuned to obtain polyphenols and antioxidants enriched extracts screening the effects of ultrasound duration on the yield of extraction and on the total phenolic content (TPC). In particular, the effects of sonication time were investigated keeping the sonicator amplitude constant (100 %, 20 kHz frequency) and varying the extraction time up to 25 min. The extraction was carried out keeping the temperature below 75 °C. Actually, though temperature conditions above 75 °C can stimulate breaking of matrix bond in addition to mass transfer phenomena, compound solubility and solvent diffusion rate, they also promote higher degradation rates of the compounds of interest [52].

The results reported in Table 1 show higher extraction efficiencies and TPC in correspondence of the longest extent of sonication (25 min). The temperature reached for this time of sonication was 71 °C and any further increment of sonication time yielded a sample temperature higher than 75 °C. In particular, the extract obtained at 25 min was characterized by an extraction yield of 7.9 % and a total phenolic content of 162 mg_GAE_/g_extract_.

**Table 1:** Values of extraction yield, TPC and technological parameters obtained for OLE at different sonication times.

Time (min)	Power (W)	T_i_ (°C)	T_f_ (°C)	ΔT (°C)	Power density (Ws/mL)	Yield (%)	TPC (mg_GAE_/g_extract_)
5	597	9	25	16	119.6	5.6 ± 0.7^A^	102 ± 5^A^
10	540	9	49	40	229.8	6.6 ± 2.3^A^	159 ± 9^B^
15	576	9	53	44	360.6	6.6 ± 1.1^A^	157 ± 4^B^
20	601	9	62	53	504.4	6.6 ± 2.2^A^	155 ± 5^B^
25	569	9	71	62	617.1	7.9 ± 1.2^A^	162 ± 2^B^

Different letters express a significant statistical difference following the Tukey’s HSD test at *p* < 0.05.

The data shows that in terms of TPC samples of 10, 15, 20 and 25 min are statistically similar. As an industrial process will be wise to use in scale up the lowest time (10 min), in this case we have chosen 25 min because reaches an higher yield and a lower SD even if are statistically similar to the other samples in the same group.

The UAE of orange peels was carried out at the Bio Based Europe Pilot Plant by a pilot scale process. Before the extraction process, matrix plant was enzymatically treated to break down pectin structure, with the aim to improve the yield of extraction and the polyphenolic contents of the extract produced. Although the extraction yield obtained for OPE is quite high, namely 39.4 %, its total phenolic content is 4 times lower than that obtained in the case of OLE.

The antioxidant capacity of both extracts was assessed by Trolox Equivalent Antioxidant Capacity (TEAC), DPPH radical scavenging assay and Ferric Ability Reducing Power (FRAP).

As reported in Table 2, the antioxidant activity evaluated by each assay is higher in the case of OLE than in the case of OPE, in agreement with the results obtained by Folin-Ciocalteu assay.

**Table 2:** Yield of extraction and antioxidant characterization of OLE and OPE.

Extract	Yield (%)	TPC (mg_GAE_/g_extract_)	TEAC (mg_TE_/g_extract_)	DPPH (mg_GAE_/g_extract_)	FRAP (mg_GAE_/g_extract_)
OLE	7.9 ± 1.2	162 ± 2	140 ± 1	44 ± 1	41 ± 3
OPE	39.4 ± 2.6	40 ± 4	83 ± 3	13 ± 2	31 ± 5

The determination of total phenolic content and antioxidant capacity is a useful tool to characterize the nature of plant extracts, however it is not sufficient to fully characterize them. Therefore, a HPLC-ESI-TOF-MS analysis was carried out to assess the polyphenolic profile of both extracts.

### 3.2 Identification and quantification of phenolic compounds by HPLC-ESI-TOF-MS

#### 3.2.1 Olive leaves extracts (OLE)

**Fig. 2:**
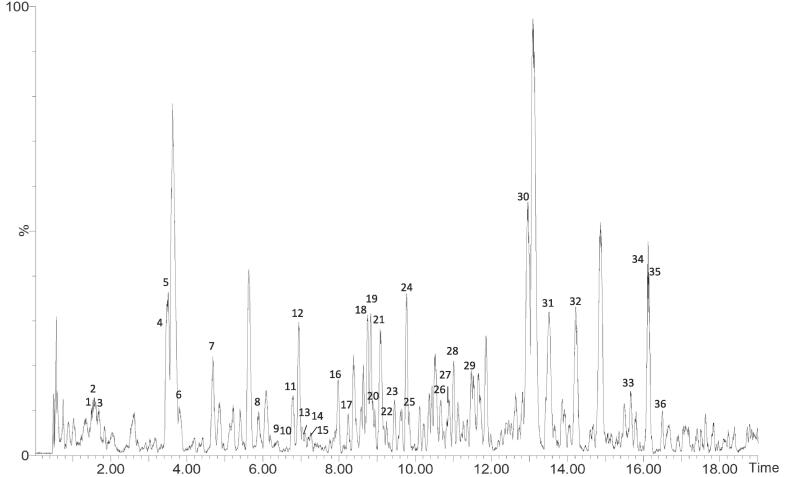
Chromatogram of OLE analyzed by HPLC-ESI-TOF-MS. Numbered peaks (1–36) correspond to the peaks reported in Table 3.

The polyphenolic profile of OLE was determined by HPLC-ESI-TOF-MS analysis, and a total of 36 compounds were identified. The results are reported in Table 3, whereas Fig. 2 shows a representative chromatogram of OLE.

**Table 3:** Identification and quantification of phenols and antioxidant compounds in OLE by HPLC-ESI-TOF-MS.

Peak	RT (min)	Compound	*m*/*z* experimental	*m*/*z* calculated	Molecular Formula	µg/g_extract_
1	1.45	Hydroxytyrosol-hexose	315.1074	315.1080	C_14_H_20_O_8_	3134.3 ± 0.2
2	1.51	Oleoside	389.1064	389.1084	C_16_H_22_O_11_	1689.6 ± 0.4
3	1.64	Hydroxytyrosol	153.0546	153.0552	C_8_H_10_O_3_	2148.9 ± 0.8
4	3.44	Oleoside/secologanoside isomer a	389.1076	389.1084	C_16_H_22_O_11_	628.7 ± 0.3
5	3.45	Oleoside/secologanoside isomer b	389.1076	389.1084	C_16_H_22_O_12_	1493.0 ± 0.5
6	3.76	Elenolic acid glucoside isomer a	403.1233	403.1240	C_17_H_24_O_11_	107.5 ± 0.1
7	4.62	Elenolic acid glucoside isomer b	403.1235	403.1240	C_17_H_24_O_11_	635.3 ± 0.1
8	5.84	Luteolin rutinoside isomer a	593.1494	593.1506	C_27_H_30_O_15_	75.5 ± 0.1
9	6.35	Elenolic acid glucoside isomer c	403.1230	403.1240	C_17_H_24_O_11_	890.4 ± 0.1
10	6.59	Dihydroxyoleuropein isomer a	571.1658	571.1663	C_25_H_32_O_15_	162.1 ± 0.1
11	6.72	Luteolin-diglucoside isomer a	609.1458	609.1456	C_27_H_30_O_16_	126.6 ± 0.1
12	6.89	Elenolic acid glucoside isomer d	403.1240	403.1240	C_17_H_24_O_11_	142.4 ± 0.1
13	7.04	β-Hydroxyverbascoside [Campneoside II] isomer a	639.1914	639.1925	C_29_H_36_O_16_	191.4 ± 0.1
14	7.19	β-Hydroxyverbascoside [Campneoside II] isomer b	639.1918	639.1925	C_29_H_36_O_16_	280.2 ± 01
15	7.52	Elenolic acid glucoside isomer e	403.1237	403.1240	C_17_H_24_O_11_	214.3 ± 0.1
16	8.16	Elenolic acid glucoside isomer f	403.1222	403.1240	C_17_H_24_O_11_	112.8 ± 0.1
17	8.35	Demethyloleuropein isomer	525.1597	525.1608	C_24_H_30_O_13_	157.0 ± 0.1
18	8.71	Hydroxyoleuropein isomer a	555.1702	555.1714	C_25_H_32_O_14_	3366.0 ± 0.2
19	8.79	Hydroxyoleuropein isomer b	555.1702	555.1714	C_25_H_32_O_14_	387.3 ± 0.1
20	9.05	Luteolin rutinoside isomer b	593.1497	593.1506	C_27_H_30_O_15_	219.2 ± 0.1
21	9.06	Luteolin glucoside isomer a	447.0918	447.0927	C_21_H_20_O_11_	356.7 ± 0.1
22	9.26	Oleuropein glucoside isomer a	701.2291	701.2293	C_31_H_42_O_18_	69.3 ± 0.1
23	9.43	Oleuropein glucoside isomer b	701.2292	701.2293	C_31_H_42_O_18_	61.6 ± 0.1
24	9.62	Hydroxyoleuropein isomer c	555.1723	555.1714	C_25_H_32_O_14_	686.8 ± 0.2
25	9.74	Verbascoside isomer a	623.1990	623.1976	C_29_H_36_O_15_	351.2 ± 0.2
26	10.72	Oleuropein glucoside isomer c	701.2292	701.2293	C_31_H_42_O_18_	717.0 ± 0.1
27	10.86	Oleuropein glucoside isomer d	701.2289	701.2293	C_31_H_42_O_18_	1049.2 ± 0.1
28	10.97	Oleuropein glucoside isomer e	701.2301	701.2293	C_31_H_42_O_18_	135.9 ± 0.1
29	11.44	Oleuropein glucoside isomer f	701.2296	701.2293	C_31_H_42_O_18_	473.2 ± 0.2
30	12.43	Hydro-oleuropein	541.1932	541.1921	C_25_H_34_O_13_	7832.1 ± 1.2
31	13.63	Ligstroside aglycone glucuronide	537.1608	537.1608	C_25_H_30_O_13_	118.7 ± 0.1
32	14.32	Luteolin	285.0399	285.0399	C_15_H_10_O_6_	253.0 ± 0.1
33	15.77	Ligstroside	523.1822	523.1816	C_25_H_32_O_12_	60.5 ± 0.1
34	16.04	Oleuropein aglycone	377.1232	377.1236	C_19_H_22_O_8_	396.4 ± 0.1
35	16.13	Frameroside/2″-epi-frameroside	601.2128	601.2132	C_27_H_38_O_15_	182.8 ± 0.1
36	16.20	Oleuroside methyl ether isomer a	553.1922	553.1921	C_26_H_34_O_13_	517.6 ± 0.1
		**Sum of oleuropein derivatives**				15493.9 ± 0.2
		**Sum of phenolic compounds**				29517 ± 2

Some of the compounds identified in the sample were classified as phenols, elenolic acid derivatives, secoiridoids and flavonoids. Most of them are glucoside derivatives due to their high presence in the matrix and to the high polarity of water employed as extracting solvent.

The amount of each compound in the sample was determined and a total of 29517 μg/g_extract_ of polyphenols was assessed, notably oleuropein derivatives represent the most abundant phenols accounting for 52.5 % of total identified phenols. Among them, hydro-oleuropein with *m*/*z* 541 is the most abundant compound (7832 μg/g_extract_). Other abundant oleuropein derivatives are hydroxyoleuropein isomers with *m*/*z* 555 and oleuropein glucoside isomers with *m*/*z* 701 (3753 μg/g_extract_ and 2506 μg/g_extract_ respectively).

Finally, OLE was found to be rich in hydroxytyrosol-hexose (*m*/*z* 315), hydroxytyrosol (*m*/*z* 153) and oleoside (*m*/*z* 389), counting for 3134 μg/g_extract_. All the other less abundant compounds are reported in Table 3.

#### 3.2.2 Orange peel extracts (OPE)

**Fig. 3:**
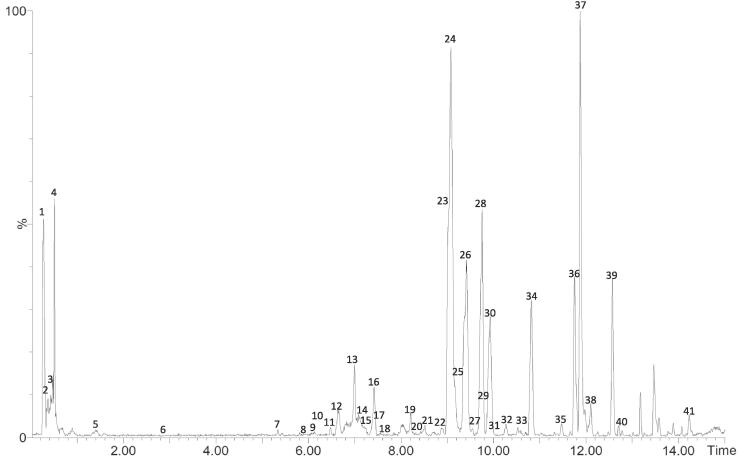
Chromatogram of OPE analyzed by HPLC-ESI-TOF-MS. Numbers 1–41 correspond to the peaks reported in Table 4.

Analogously to OLE, phenolic compounds present in OPE were characterized by HPLC-ESI-TOF-MS analysis. A representative chromatogram of OPE is reported in Fig. 3.

Table 4 reports the 41 polar compounds identified in OPE, in good agreement with a previous report [53], among them only phenolic acids and flavonoids were quantified.

**Table 4:** Identification and quantification of phenols and antioxidant compounds in OPE by HPLC-ESI-TOF-MS.

Peak	RT (min)	Compound	*m*/*z* experimental	*m*/*z* calculated	Molecular formula	µg/g_extract_
1	0.36	Gluconic acid isomer a	195.0499	195.0505	C_6_H_12_O_7_	–
2	0.39	Citric acid	191.0185	191.0192	C_6_H_8_O_7_	–
3	0.43	Gluconic acid isomer b	195.0498	195.0505	C_6_H_12_O_7_	–
4	0.49	Isocitric acid	191.0183	191.0192	C_6_H_8_O_7_	–
5	1.61	Norbergenin	313.0548	313.0560	C_13_H_14_O_9_	144.3 ± 0.1
6	2.85	Caffeoylglycolic acid methyl ester isomer a	251.0552	251.0556	C_12_H_12_O_6_	1355.0 ± 11.5
7	5.38	Cyranoside A	443.1900	443.1917	C_21_H_32_O_10_	729.9 ± 2.0
8	5.88	Caffeoylglycolic acid methyl ester isomer b	251.0547	251.0556	C_12_H_12_O_6_	1142.3 ± 10.0
9	6.14	Caffeoylmalic acid isomer a	295.0441	295.0454	C_13_H_12_O_8_	1330.3 ± 11.0
10	6.20	Citroside	385.1845	385.1862	C_19_H_30_O_8_	–
11	6.58	Rutin	609.1436	609.1456	C_27_H_30_O_16_	< LOQ
12	6.64	Apigenin-di-C-hexoside (Vicenin-2) isomer a	593.1532	593.1506	C_27_H_30_O_15_	1114.3 ± 7.1
13	7.00	Apigenin-di-C-hexoside (Vicenin-2) isomer b	593.1534	593.1506	C_27_H_30_O_15_	3283 ± 14
14	7.18	Dihydroisorhamnetin 7-rutinoside	625.1798	625.1827	C_21_H_38_O_21_	25.1 ± 0.7
15	7.37	Isorhamnetin-3-O-rutinoside isomer a	623.1586	623.1612	C_28_H_32_O_16_	50.2 ± 3.1
16	7.51	Isorhamnetin-3-O-rutinoside isomer b	623.1613	623.1612	C_28_H_32_O_16_	102.4 ± 2.8
17	7.51	Caffeoylmalic acid isomer b	295.0449	295.0454	C_13_H_12_O_8_	1126.1 ± 10.1
18	7.65	Isorhamnetin-3-O-rutinoside isomer c	623.1597	623.1612	C_28_H_32_O_16_	< LOQ
19	8.36	Alpha-glucosyl hesperidin	771.2352	771.2348	C_34_H_44_O_20_	560.3 ± 2.2
20	8.40	Eriocitrin	595.1657	595.1663	C_27_H_32_O_15_	< LOQ
21	8.61	Vitexin-O-pentoside isomer a	563.1392	563.1401	C_26_H_28_O_14_	315.3 ± 1.6
22	8.81	Naringin hydrate	597.1835	597.1819	C_27_H_34_O_15_	202.8 ± 2.7
23	9.00	Vitexin-O-pentoside isomer b	563.1400	563.1401	C_26_H_28_O_14_	467.2 ± 4.7
24	9.08	Limonin 17-β-D-glucopyranoside	649.2471	649.2496	C_32_H_42_O_14_	–
25	9.17	Prunin	433.1132	433.1135	C_21_H_22_O_10_	1634.9 ± 2.4
26	9.18	Naringenin	271.0599	271.0606	C_15_H_12_O_5_	1473.7 ± 1.2
27	9.35	Naringin 4′-glucoside	741.2255	741.2242	C_33_H_42_O_19_	144.2 ± 3.3
28	9.41	Narirutin isomer a	579.1708	579.1714	C_27_H_32_O_14_	7319.6 ± 11.8
29	9.55	Kaempferol 3-rhamnoside-7-galacturonide	607.1310	607.1299	C_27_H_28_O_16_	89.1 ± 4.4
30	9.75	Narirutin isomer b	579.1722	579.1714	C_27_H_32_O_14_	7337.7 ± 57.1
31	9.89	Hesperetin 7-O-glucoside	463.1244	463.1240	C_22_H_24_O_11_	174.7 ± 0.9
32	10.24	Hesperidin	609.1849	609.1819	C_28_H_34_O_15_	3051.7 ± 25.9
33	10.58	Isorhamnetin-3-O-rutinoside isomer d	623.1661	623.1671	C_21_H_35_O_21_	< LOQ
34	10.82	Isoobacunoic acid 17-β-D-glucoside	651.2642	651.2653	C_32_H_44_O_14_	–
35	11.38	Pectolinarin	621.1833	621.1819	C_29_H_34_O_15_	< LOQ
36	11.79	Didymin isomer a	593.1882	593.1870	C_28_H_34_O_14_	579.7 ± 4.0
37	11.88	Nomilin 17-O-β-D-glucopyranoside	693.2768	693.2758	C_34_H_45_O_15_	–
38	12.11	Didymin isomer b	593.1869	593.1870	C_28_H_34_O_14_	333.8 ± 6.1
39	12.57	Nomilinic acid 17-β-D-glucoside	711.2861	711.2864	C_34_H_48_O_16_	–
40	12.71	Obacunone 17-β-D-glucoside	633.2568	633.2547	C_32_H_42_O_13_	–
41	14.23	Limonin	469.1854	469.1862	C_26_H_30_O_8_	–
		**Sum of phenolic acids**				5098 ± 42
		**Sum of flavonoids**				28977 ± 76
		**Sum of total phenolic compounds**				34075 ± 118

For what concerns flavonoids, narirutin isomers (*m*/*z* 579) are the most abundant phenols in OPE, corresponding to 14658 μg/g_extract_; then, in order of abundance, vicenin-2 isomers (*m*/*z* 593), hesperidin (*m*/*z* 609), prunin (*m*/*z* 433) and naringenin (*m*/*z* 271) count for 4397 μg/g_extract_, 3052 μg/g_extract_, 1635 μg/g_extract_ and 1474 μg/g_extract_, respectively.

For what concerns phenolic acids, the main compounds quantified are caffeoylglycolic acid methyl ester isomers (*m*/*z* 251) and caffeoylmalic acid isomers (*m*/*z* 295), 2497 μg/g_extract_ and 2456 μg/g_extract_ respectively.

### 3.3 Preparation and characterization of liposomes

#### 3.3.1 Preparation of liposomes

With the aim of protecting the OLE and OPE from physical and biological degradation and deliver them with high efficiency to the target bacteria, we investigated their inclusion into liposomes formulated with a natural unsaturated phospholipid (DOPC) and cholesterol (Chol), at a 8:2 DOPC/Chol ratio and total lipid concentration of 10 mM. The presence of Chol in the formulation involves a more compact and stable lipid membrane with reduced permeability to water-soluble compounds, thus increasing the retention of the entrapped cargo [54].

#### 3.3.2 Size and ζ-potential determination

The mean diameter, the polydispersity index (PDI) and the ζ-potential values of empty and loaded DOPC/Chol liposomes were investigated and the results are reported in Table 5.

**Table 5:** Hydrodynamic diameter (D_h_), PDI, ζ-Potential and Entrapment Efficiency (EE%) of empty and loaded liposomes (10 mM in total lipids) in PBS (pH 7.4).

Composition	D_h_ (nm)	PDI	ζ-Potential (mV)	EE (%)
**DOPC/Chol** 8.0:2.0	95 ± 1	0.25 ± 0.01	−2.7 ± 0.6	–
**DOPC/Chol/OLE** 8.0:2.0	96 ± 1	0.21 ± 0.01	−4.5 ± 0.9	29 ± 5
**DOPC/Chol/OPE** 8.0.2.0	101 ± 1	0.22 ± 0.01	−5.3 ± 0.5	11 ± 3

As shown in Table 5 all formulations show monomodal size distributions characterized by dimensions ranging between 95 nm and 101 nm. The presence of OPE in the liposomes induces a slight increase of hydrodynamic diameter with respect to empty liposomes. This suggests that loaded compounds induce a different organization of lipid membrane, thus modifying its properties [55]. The PDI values of all the systems, in the range 0.21–0.25, reveal the homogeneity and uniformity of the investigated liposomes.

The values of ζ-potential of liposomes loaded either with OLE or with OPE are lower with respect to empty liposomes, thus suggesting that the extract compounds are partially localized at the lipid/water interface thus changing the net surface charge of liposomes. The difference in ζ-potential values of OLE and OPE loaded liposomes are due to the different nature of encapsulated phenolic compounds and to their amount absorbed at the surface of liposome membrane.

#### 3.3.3 Entrapment efficiency of extracts

The Entrapment Efficiencies (EE%) of OLE and OPE loaded into liposomes were assessed by Folin–Ciocalteu assay. Following this procedure, the amount of total polyphenols entrapped in DOPC/Chol liposomes was evaluated in comparison with their amount present in the free extracts. As reported in Table 5, the EE% measured for OLE and OPE was 29 % and 11 %, corresponding to 302 μg_GAE_/mL and 40 μg_GAE_/mL, respectively. Therefore, in the case of OLE, the amount of total polyphenols entrapped into liposomes is more than seven times higher than in the case of OPE. Although the EE% found in the case of OLE might seem low, the quantity of encapsulated phenols is fairly high. On the other hand, the low amount of polyphenols encapsulated into liposomes in the case of OPE could be due to the more hydrophilic nature of the polyphenolic compounds present in OPE.

#### 3.3.4 Stability to storage

**Fig. 4:**
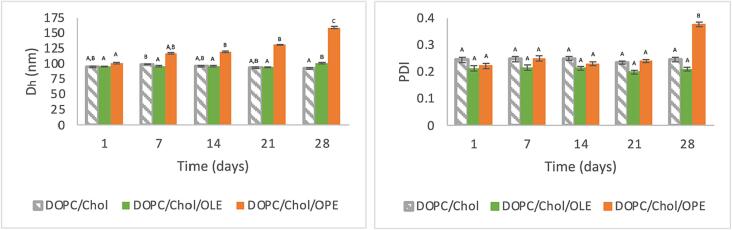
Liposome particle size diameter (D_h_) and PDI values during 28 days of storage at 4 °C in the dark. Different letters express a significant statistical difference following the Tukey’s HSD test at p < 0.05.

In order to investigate the physical stability of empty and loaded liposomes, particle hydrodynamic diameter and PDI values were evaluated by DLS measurements over 28 days of storage at 4 °C protected from light sources. As shown in Fig. 4, the size and PDI of liposomes during storage statistically changed only in the case of DOPC/Chol/OPE formulation at 28 days. In fact, in this case a progressive increase of dimensions, from 101 nm to 159 nm, and an increment of PDI value was observed at 28 days as well. The increase of nanoparticles size could be due to vesicle aggregation phenomena [56], [57].

#### 3.3.5 *In vitro* release study

To evaluate the ability of liposomes to act as extract delivery systems, an *in vitro* release study was carried out using dialysis. The release over time of phenolic compounds from DOPC/Chol/OLE and DOPC/Chol/OPE liposomes was evaluated from dialyzed samples by Folin-Ciocalteu assay, determining the total phenolic content still encapsulated in liposomes over a period of 24 h.

**Fig. 5:**
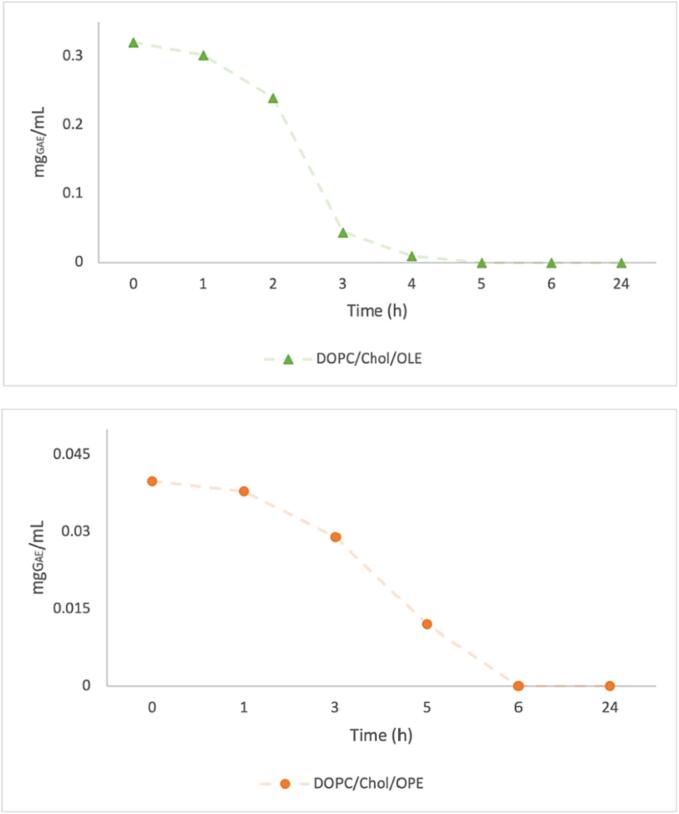
*In vitro* forced release of OLE (green triangles) and OPE (orange dots) from DOPC/Chol liposomes. (For interpretation of the references to colour in this figure legend, the reader is referred to the web version of this article.)

As shown in Fig. 5, 80:20 DOPC/Chol liposomes release 50 % of entrapped polyphenols within 2–3 h in the case of OLE and within 3–4 h in the case of OPE, with a complete cargo release in 5 h for OLE and 6 h for OPE.

### 3.4 Antimicrobial activity

In the present study the antimicrobial activity of OLE and OPE, either free or loaded in DOPC/Chol liposomes, was investigated by the broth macrodilution method. Firstly, the antimicrobial activity of free OLE and OPE was screened against six different strains of potential bacterial pathogenic species, three *Gram*-positive and three *Gram*-negative. The tested microorganisms showed a variable susceptibility to OLE and OPE as reported in Table 6.

**Table 6:** Susceptibility of bacterial pathogen strains to OLE and OPE.

	*Gram*-positive			*Gram*-negative		
Extract	*S. aureus* NCIMB 9518	*E. faecalis* NCIMB 775	*B. subtilis* ATCC 6051	*E. coli* NCIMB 13302	*K. oxytoca* NCIMB 12259	*P. aeruginosa* NCIMB 9904
OLE	+	–	–	–	–	–
OPE	–	–	+	–	–	–

+ effective - not effective.

Both OLE and OPE did not show any antimicrobial activity against bacteria species belonging to the screened *Gram*-negative strains. Although some examples are reported in the literature in which orange peels and olive leaves extracts have exerted antimicrobial activities against specific strains of *Gram*-negative bacteria, the activity of these extracts is tightly related to their polyphenolic profile, which can vary depending on the solvent and the technique used for their preparation, as well as for the type of cultivar from which they were obtained. Moreover, it is worth of note that generally the treatment of *Gram*-negative bacterial infections is more difficult because of the presence of active efflux pumps, of the production of antibiotic degrading enzymes and of some additional resistance mechanisms to antibiotics due to the structure of the outer membrane of these bacteria, composed by lipopolysaccharide and proteins; all these factors influence and reduce their susceptibility to various antimicrobial drugs [58], [59].

On the other hand, OLE was found to be selectively effective against a *Gram*-positive pathogen strain, namely *S. aureus,* with a MIC value of 7 mg/mL corresponding to 1.135 mg_GAE_/mL (as assessed by Folin-Ciocalteu assay, see Table 7) and OPE showed an antimicrobial activity against *B. subtilis* with a MIC value of 10 mg/mL corresponding to 0.403 mg_GAE_/mL (as assessed by Folin-Ciocalteu assay, see Table 7). In both cases, MLC values were not determined because it was considered not relevant and useful to test extract concentrations higher than 10 mg/mL.

**Table 7:** Minimum Inhibitory Concentration (MIC) of OLE and OPE on *S. aureus* (NCIMB 9518) and *B. subtilis* (ATCC 6051) bacteria, MIC values are reported both as milligrams of extract per milliliter (mg/mL) and as milligrams of gallic acid equivalents per milliliter (mg_GAE_/mL, assessed by Folin-Ciocalteu assay).

MIC					
*S. aureus* (NCIMB 9518)			*B. subtilis* (ATCC 6051)		
Extract	mg/mL	mg_GAE_/mL	Extract	mg/mL	mg_GAE_/mL
OLE	7	1.135	OPE	10	0.403

Liposomes can protect polyphenols from chemical and biological degradation [60], further they can be a useful tool to deliver them efficiently to a specific tissue or cell target, also eluding specific mechanisms of resistance [61], therefore we investigated the antimicrobial activity of OLE and OPE included into DOPC/Chol liposomes. Because we ascribe the antimicrobial activity of the extracts to the polyphenols and we cannot quantify their total amount when encapsulated, we assumed as reasonable to report MIC and MLC values of both free (see above) and encapsulated extracts as milligrams of gallic acid equivalents per milliliter (mg_GAE_/mL) in order to have values useful for the comparison. OLE loaded in liposomes showed an antimicrobial activity with a final MIC value of 0.113 mg_GAE_/mL against *S. aureus*; experimentally we couldn’t determine MLC, in fact we evaluated that it is higher than 0.151 mg_GAE_/mL, which was the highest concentration testable. Therefore, by comparing the MIC values of OLE tested in free form and loaded in liposomes (see Table 8), it is worth of note that the encapsulation of OLE in liposomes showed a positive effect on the activity against *S. aureus* by increasing the antimicrobial activity of OLE encapsulated by ∼ 10 times. This great effect could be related to the surface polarity of liposomes that enhances the interaction with bacteria membrane surface. This could lead to the better diffusion and interaction of the active compounds released from the lipid bilayer across the bacterial cell walls, favouring their permeability and affecting bacteria organelles, eventually resulting in the inhibition of bacterial growth [62]. Therefore, the inclusion of OLE polyphenols in liposomes not only increases their solubility in biological fluids, their bioavailability at the target sites and the protective effect from internal and external degradation by retarding chemical reactions [63], [64], and improves its antimicrobial activity.

**Table 8:** Comparison between MIC values, reported as milligrams of gallic acid equivalents per milliliter (mg_GAE_/mL), obtained for OLE in free form and loaded in DOPC/CHOL liposome on *S. aureus* (NCIMB 9518).

	MIC (mg_GAE_/mL)
OLE in free form	1.135
DOPC/Chol/OLE	0.113
DOPC/Chol	n.a.

n.a. = no active.

On the other hand, the inclusion of OPE in DOPC/Chol liposomes did not show the same beneficial effect observed for OLE in terms of antimicrobial activity. In fact, it was not possible to assess MIC and MLC values of encapsulated OPE against *B. subtilis*, which are certainly higher than the highest testable concentration. This is due to the EE% obtained for DOPC/Chol/OPE liposomes corresponding to 11 % of total OPE polyphenols, which was not sufficient to achieve any inhibitory effects.

The antimicrobial activity of DOPC/Chol empty liposomes was evaluated against the bacterial strains responsive to the action of OLE and OPE, *S. aureus* (NCIMB 9518) and *B. subtilis* (ATCC 6051). In both cases there was no evidence of antimicrobial activity caused by the lipidic components of liposomes. Therefore, the activity observed in the case of OLE loaded DOPC/CHOL liposomes against *S. aureus* (NCIMB 9518) is exclusively to ascribe to the encapsulated OLE polyphenols.

## 4 Conclusions

Olive leaves and orange peels are good sources of phenolic compounds with high benefits to human health due to their antioxidant, antibacterial and antiproliferative activities.

In this work we obtained extracts from olive leaves and orange peels, rich in polyphenolic compounds by UAE using a food-grade solvent, such as water. Extracts were characterized in terms of total phenolic content and antioxidant capacity, moreover their polyphenolic profile was investigated by HPLC-ESI-TOF-MS analysis.

The efficient encapsulation of extracts into liposomes formulated with a natural phospholipid (DOPC) and cholesterol, beside enhancing the solubility, stability and then bioavailability of the loaded phenols proved to improve their antimicrobial activity. In particular, the encapsulation of OLE in DOPC/Chol liposomes enhances its antibacterial activity against *S. aureus* by an order of magnitude.

## CRediT authorship contribution statement

**Giuliana Prevete:** Data curation, Investigation, Methodology, Validation, Visualization, Writing – original draft. **Loïc G. Carvalho:** Funding acquisition, Investigation, Methodology, Resources, Validation, Writing – review & editing. **Maria del Carmen Razola-Diaz:** Data curation, Investigation, Methodology, Validation, Writing – review & editing. **Vito Verardo:** Data curation, Funding acquisition, Investigation, Methodology, Validation, Writing – review & editing. **Giovanna Mancini:** Conceptualization, Funding acquisition, Writing – review & editing. **Alberto Fiore:** Conceptualization, Funding acquisition, Writing – review & editing. **Marco Mazzonna:** Conceptualization, Data curation, Funding acquisition, Supervision, Visualization, Writing – original draft.

## Declaration of competing interest

The authors declare that they have no known competing financial interests or personal relationships that could have appeared to influence the work reported in this paper.
